# Velocity measurements in whole blood using acoustic resolution photoacoustic Doppler

**DOI:** 10.1364/BOE.7.002789

**Published:** 2016-06-23

**Authors:** Joanna Brunker, Paul Beard

**Affiliations:** 1 Department of Medical Physics and Biomedical Engineering, University College London, Gower Street, London, WC1E 6BT, UK; 2 joanna.brunker.09@ucl.ac.uk; 3 paul.beard@ucl.ac.uk

**Keywords:** (280.2490) Flow diagnostics, (110.6150) Speckle imaging, (170.5120) Photoacoustic imaging, (170.7170) Ultrasound, (110.4153) Motion estimation and optical flow, (110.5125) Photoacoustics

## Abstract

Acoustic resolution photoacoustic Doppler velocimetry promises to overcome the spatial resolution and depth penetration limitations of current blood flow measuring methods. Despite successful implementation using blood-mimicking fluids, measurements in blood have proved challenging, thus preventing *in vivo* application. A common explanation for this difficulty is that whole blood is insufficiently heterogeneous relative to detector frequencies of tens of MHz compatible with deep tissue photoacoustic measurements. Through rigorous experimental measurements we provide new insight that refutes this assertion. We show for the first time that, by careful choice of the detector frequency and field-of-view, and by employing novel signal processing methods, it is possible to make velocity measurements in whole blood using transducers with frequencies in the tens of MHz range. These findings have important implications for the prospects of making deep tissue measurements of blood flow relevant to the study of microcirculatory abnormalities associated with cancer, diabetes, atherosclerosis and other conditions.

## 1. Introduction

Photoacoustic flowmetry (PAF) is an emerging technique that offers significant advantages compared to existing methods for measuring blood velocity. The general principle of PAF involves illuminating red blood cells (RBCs) with modulated laser light in order to generate ultrasound (photoacoustic) waves; the motion of the RBCs is then monitored via changes such as time, phase or frequency shifts in the photoacoustic waves they emit. Unlike Doppler ultrasound [1] photoacoustic methods are well suited to measuring the low flow velocities in microvessels. This is a result of the high optical absorption difference between blood and the surrounding tissue, which enables superior blood vessel contrast; in Doppler ultrasound the small signal backscattered from microvessels is difficult to distinguish from the surrounding tissue which may also be moving at comparable speeds due to respiratory and cardiac motion. In addition, when using diffuse illumination, PAF has several advantages over purely optical techniques. It can provide greater penetration depth than optical methods such as flow cytometry [2], confocal microscopy [3] and optical coherence tomography [4,5] that use ballistic photons and are therefore limited to depths of less than 1 millimetre due to optical scattering. Deeper penetration is possible using optical techniques such as laser Doppler velocimetry [6] that employ diffuse light rather than ballistic photons but resolution is relatively poor. By contrast PAF can provide better spatial resolution on account of the weaker scattering of acoustic waves compared to light.

Various photoacoustic flowmetry methods have been developed and successfully validated using blood flow phantoms [7–12]. However, *in vivo* velocity measurements [13–18] have so far only been achieved by focussing the laser light in the tissue. This so-called “optical resolution” (OR) mode is limited to a maximum penetration depth of less than approximately 1 mm due to strong optical scattering in tissue, and thus fails to exploit the intrinsic potential of photoacoustic velocimetry for deeper tissue measurements. The “acoustic resolution” (AR) mode employs wide-field diffuse illumination and the signal is localised by defining the region from which the photoacoustic waves are received by using a focussed ultrasound detector or array. The use of diffuse illumination opens up the possibility for several centimetres of tissue penetration, but the successful implementation of AR-PAF remains a challenge.

Previous work [19,20] has demonstrated the underlying physical principles of AR-PAF using blood-mimicking phantoms comprising micron scale absorber suspensions, but only at concentrations an order of magnitude lower than a physiologically realistic RBC haematocrit. For higher concentrations it has proved very challenging to make accurate velocity measurements [19]. Perhaps unsurprisingly therefore AR-PAF measurements in whole blood *in vivo* have not been reported either. A common explanation for this difficulty is that whole blood is insufficiently optically heterogeneous to permit accurate AR-PAF measurements using detector frequencies consistent with mm-cm scale penetration depths in tissue (i.e. frequencies in the MHz to tens of MHz range). Optical heterogeneity is a crucial requirement for the detection of flow using AR-PAF as without it moving blood resembles a continuum that is photoacoustically indistinguishable from static blood. To perceive heterogeneity requires an ultrasound detector able to resolve the “granularity” of blood; however, a simple consideration of the spatial scale of the distribution of RBCs in whole blood suggests that adequate resolution would only be provided by detector frequencies well beyond the tens of MHz range. Through experimental AR-PAF measurements in whole blood we provide new insight and evidence that refutes this assertion.

This study shows for the first time that, by careful choice of the detector bandwidth and field-of-view, and by employing novel signal processing methods, it is possible to make AR-PAF measurements in whole blood using detectors with frequencies in the tens of MHz range. The requirement for heterogeneity in the blood is investigated using detectors with different centre frequencies, and also different degrees of RBC heterogeneity. In addition, different wavelengths of light and a range-gating analysis are employed to validate and address the light penetration problem identified in previous work [19]. These findings have significant implications for the prospects of making deep tissue measurements of blood flow relevant to the study of microcirculatory abnormalities associated with vascular diseases [21], cancer [22] and other conditions.

## 2. Time correlation acoustic resolution photoacoustic Doppler velocimetry

**Fig. 1:**
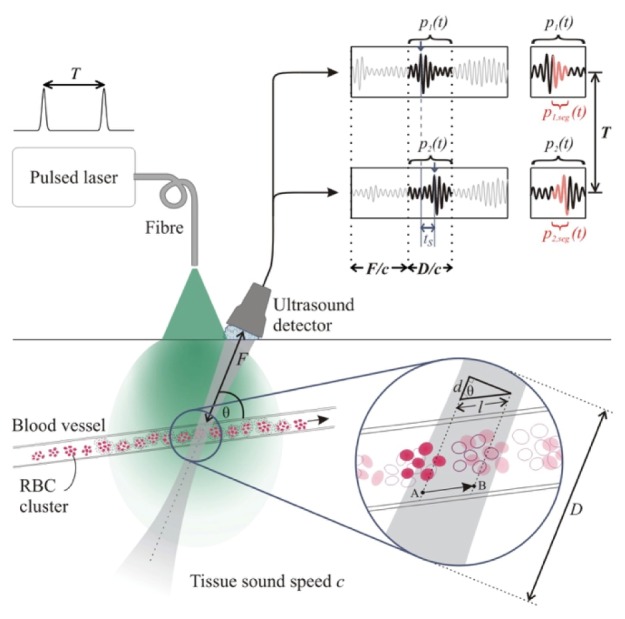
Schematic illustrating the principle of time correlation AR-PAF. The two signals are generated from clusters of moving red blood cells (RBCs) when illuminated by a pair of laser pulses separated by a time *T*. The inset shows the distribution of RBCs (represented by solid ellipses) when the first laser pulse is fired, and the new positions (unfilled ellipses) coincident with the firing of the second laser pulse a time *T* later. Between the two pulses the RBCs have moved from *A* to *B*, a distance *l* along the blood vessel. The waveforms show sections of the two photoacoustic signals *p*_1_(*t*) and *p*_2_(*t*) that correspond to the location of the blood vessel and illustrate the time shift *t*_s_ between the two due to the motion of the RBCs along the vessel. It is also possible to calculate the velocity in specific regions of the vessel by time-windowing to select appropriate signal segments such as *p*_1,seg_(*t*) and *p*_2,seg_(*t*) in a manner analagous to range-gating in Doppler ultrasound.

The principles of acoustic resolution photoacoustic Doppler velocimetry are described in detail in references [7,19,23], and the concept is illustrated in Fig. 1

. Briefly, wide-field illumination of the tissue using a pair of laser pulses separated by a time *T* produce a corresponding pair of photoacoustic waves emitted by RBCs flowing in a subsurface vessel. Upon detection at the surface, these waves gives rise to a pair of photoacoustic waveforms that are identical but time shifted with respect to each other due to the motion of the RBCs. The time shift *t*_s_ is estimated by cross-correlation of the two waveforms and used to calculate the RBC velocity *V’*:

$$V'=\frac{ct_{s}}{T\cos\theta }$$ (1)

where *c* is the sound speed, and θ is the angle between the transducer axis and the flow direction. As illustrated in Fig. 1, the measurement is localised by selecting an appropriate time-window of each waveform in a manner analogous to range-gating in conventional pulsed wave Doppler ultrasound. So, for example, with reference to Fig. 1, *p*_1_(*t*) and *p*_2_(*t*) of the detected waveforms are segments that correspond to the location of the vessel at a distance *F* along the transducer axis and the length *D* of these segments defines the axial spatial resolution. If *D* is larger than the blood vessel diameter projected along the transducer axis, the measurement is integrated over the vessel cross section and the estimated time shift *t*_s_ yields the average velocity of the RBCs. Time-windowing to select a smaller segment of the signals, giving for example *p*_1,seg_(*t*) and *p*_2,seg_(*t*), enables estimation of the velocity at a specific range-gate location along the transducer axis within the vessel. In each case the time shifts are estimated by calculating the cross-correlation between the corresponding signal segments [7,19].

## 3. Experimental design

Figure 2

**Fig. 2:**
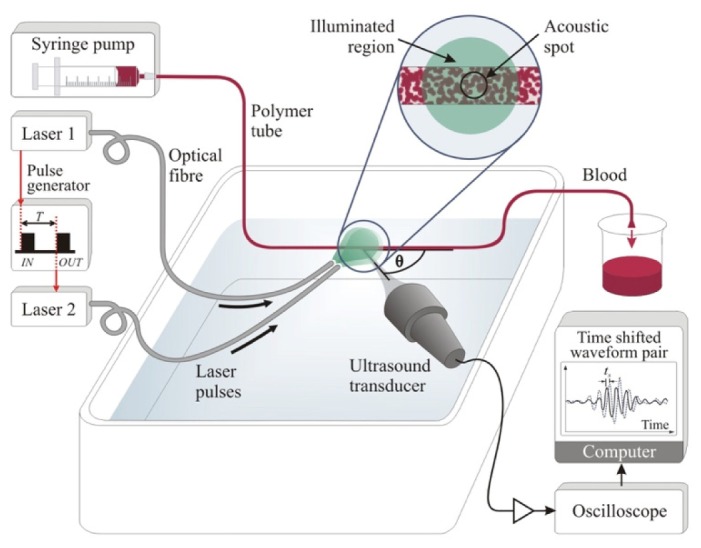
Experimental setup for pulsed photoacoustic Doppler blood flow measurements. Laser pulses separated by a time *T* are used to generate pairs of photoacoustic waveforms which are detected by an ultrasound receiver positioned at an angle θ to the flow axis. This angle was measured to the nearest degree using a turntable with angular markings at 1° intervals, and verified by horizontally translating the tube and comparing the measured distances with those calculated from cross-correlation of photoacoustic signals acquired before and after translation. The inset shows that whilst a large area (at least 5 mm diameter) of the red blood cells (RBCs) is illuminated, photoacoustic signals are collected from a smaller region defined by the transducer focal spot in order to be representative of the acoustic resolution mode of photoacoustic detection.

shows the experimental setup used to acquire photoacoustic blood velocity measurements using excitation wavelengths in the range 450 nm to 680 nm. In principle, the measurements could be achieved by using a single laser source to generate a pair of laser pulses. However, in the absence of a wavelength-tuneable laser system capable of providing nanosecond laser pulses with the required sub-millisecond pulse separations, two OPO laser systems (Innolas Spitlight 600 and Newport Spectra-Physics Quanta-Ray / GWU GmbH VisIR) were used. The two laser systems were sequentially triggered via a pulse delay generator which was set to deliver an appropriate time delay (*T* = 0.5 ms) between successive laser pulses in a pair. The output of both lasers were coupled into 1.5 mm diameter multimode optical fibres positioned so as to illuminate the same area (approximately 1 cm^2^, with a fluence < 10 mJ/cm^2^) of the polymer tube (diameter approximately 0.4 mm) containing flowing RBCs. The photoacoustic signals were detected using one of the six focussed ultrasound detectors listed in Table 1

**Table 1:** Spherically focussed transducers used variously to detect photoacoustic signals generated in red blood cells*^a^*

Centre frequency, f_0_		−6 dB bandwidth*^a^*		Transducer material*^b^*		Focal length		FWHM beam width*^c^*		Axial resolution*^a^*
MHz		MHz				mm		mm		mm
5		2		PZT		64		0.97*		0.60
15		8		PZT		19		0.15		0.20
20		11		PZT		19		0.33*		0.15
30		23		PZT		19		0.35*		0.06
50		40		PZT		51		0.24		0.04
25		25		PVDF		24		0.24		0.07

^a^ Estimated from the manufacturers’ specifications.

^b^ The PZT transducers were manufactured by Panametrics Olympus NDT Inc. (Waltham, Massachusetts, USA), and the 25 MHz transducer was made by Precision Acoustics Ltd. (Dorchester, UK).

^c^ Values of the full-width-half-maximum (FWHM) at the transducer focus: the FWHM values marked * were measured by mapping the transmitted transducer beam profiles using a Fabry-Perot sensor [24]; the other FWHM values are calculated from the transducer characteristics using Eq. (9) in ref [25].

. The signals were amplified and captured by an oscilloscope, and then downloaded to a PC, where they were processed to calculate the time shift *t_s_* that arises due to motion of the RBCs. The diameter of the illuminated region was at least 5 mm and thus much larger than the lateral dimensions of the detector focus in order to be representative of the acoustic resolution mode of photoacoustic detection.

**Fig. 3:**
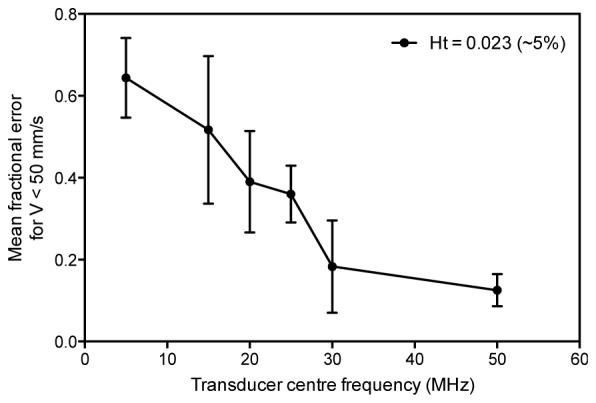
Comparison of the accuracy of velocity measurements made using transducers with centre frequencies ranging from 5 MHz to 50 MHz (see Table 1). For each transducer the measurements were made for red blood cells (5% of a normal physiological haematocrit) flowing in a 390 μm tube, and illuminated with 532 nm laser pulses separated by *T* = 0.5 ms. The accuracies are the fractional errors of measurements *V*’ made for known velocities *V* < 50 mm/s. The error bars represent the standard error on the mean fractional error.

Velocity measurements were made for various blood samples at different concentrations expressed as a percentage of the undiluted sample, which had a physiologically representative concentration. A typical concentration for the 100% (undiluted) case was 5 x 10^9^ RBCs per ml (haematocrit: 0.56; haemoglobin concentration: 155 g/l) but this varied between subjects; the specific measured concentrations are therefore stated in the figures for each result in section 4. For the measurements shown in section 4.3, the RBCs were sourced from whole human blood at a normal physiological concentration and diluted to 25% and 12.5% using phosphate buffered saline (PBS). The samples were refrigerated when not in use, and all experiments were conducted within nine days of obtaining the fresh sample from the human volunteer. In order to produce the range of haematocrits shown in Fig. 5 the human blood samples were first washed with PBS and then diluted to concentrations ranging from 3% to 80%, and the haematocrits verified in each case (Sysmex XE-5000 Automated Hematology System). For the measurements shown in Fig. 3

the erythrocytes (red blood cells, RBCs) were obtained from the UCLH Blood Transfusion Unit in additive solution, with the leucocytes (white blood cells) depleted. Phosphate buffered saline was added to give a concentration of RBCs equivalent to approximately 5% of a normal haematocrit.

To obtain a single velocity estimate, the tube was irradiated by a series of 25 laser pulse pairs. The pulses within each pair were separated by *T* = 0.5 ms, which was identified as optimal for the range of velocities investigated. Measurements *V’* ± Δ*V’*/2 were made for average flow speeds |*V*| in the range 0 to 80 mm/s. The syringe pump could be programmed to deliver flow rates in steps of 0.01 ml/hr, and the pre-selected rate and the inner diameter of the tube were used to calculate |*V*| in mm/s. Uncertainties Δ*V* were based on the 5% tolerance in the diameter of the tubing. These “known” values and uncertainties correspond to the average flow velocity *V* ± Δ*V*/2 and were compared with the measured *V’* ± Δ*V’*/2 acquired via cross-correlation of the photoacoustic waveform pairs, as described in references [7,19]. The average flow velocity was calculated by cross-correlating the entire 5000-point (1.25 µs) time series of the signals, or by applying the range-gating method which involved evaluating signal segments in 250-point (62.5 ns) time windows, as described in reference [19] and in the previous section.

## 4. Results: blood velocity measurements

Previous work [19] has demonstrated the feasibility of making velocity measurements in a blood mimicking phantom comprising an aqueous suspension of 3 µm polystyrene microspheres using time correlation AR-PAF. However, it was observed that for concentrations approaching physiologically realistic levels, the measurement accuracy and resolution were poor. Indeed at the highest concentrations used it was impossible even to detect flow. Two factors were identified as being responsible for these limitations. The first relates to the requirement for sufficient absorber heterogeneity as perceived by the detector. At high concentrations, the finite bandwidth of the detector and its lateral field-of-view (FOV) can result in the absorber distribution being perceived as optically homogenous thereby precluding the detection of flow. The second factor relates to the extent to which light can penetrate into the vessel. Insufficient light penetration can lead to measurements that are biased towards the slower velocities at the edge of the vessel resulting in under-reading of the average flow velocity. In the following sections, both factors are explored using blood as the absorber, rather than the absorbing microspheres used previously [19]. Sections 4.1 and 4.2 describe the influence of the detector characteristics in the context of absorber heterogeneity and their impact on velocity measurement accuracy. The results in Section 4.3 illustrate how velocity measurement accuracy varies for different RBC concentrations, and using different wavelengths of light in order to vary the light penetration.

### 4.1 Absorber heterogeneity: photoacoustic signal bandwidth and the influence of the detector characteristics

The critical challenge in terms of making accurate AR-PAF measurements in whole blood relates to the spatial scale of the distribution of the RBCs within the plasma relative to the minimum detectable acoustic wavelength. More generally, this is referred to as the degree of absorber “heterogeneity”. Some level of spatial heterogeneity in the absorption distribution is essential. This is because time-correlation AR-PAF relies upon tracking the motion of a cluster of RBCs by measuring time shifts in the photoacoustic signals emitted by the cluster when irradiated by a pair of laser pulses. If the RBCs are so tightly packed as to approximate an optically homogeneous medium, the photoacoustic waveforms produced by successive laser pulses will be temporally identical. There is therefore no detectable time shift and the motion is indistinguishable from the static case. “Heterogeneity” in this context is very much a relative term. It depends critically on the ability of the detector to resolve the “granularity” of the absorber distribution. This resolving ability is a function of both the intrinsic bandwidth of the detector and its lateral field-of-view as discussed below.

#### 4.1.1 Intrinsic detector centre frequency and bandwidth

Absorber heterogeneity depends most obviously on the detector centre frequency and bandwidth. An ensemble of microscopic absorbers that is perceived as heterogeneous using a broadband high frequency detector may appear homogenous to a lower frequency detector and preclude the detection of flow. In order to illustrate this notion of perceived heterogeneity, velocity measurements of a RBC suspension flowing in a 390 µm tube were made using the six different transducers in Table 1. In these experiments, a relatively low concentration of RBCs was used (haematocrit Ht = 0.023, approximately 5% of a physiologically normal haematocrit) for illustrative purposes. Figure 3 shows the accuracy obtained for the different transducers, plotted as the mean fractional error between the known *V* and measured *V’* average velocities for all *N* measurements made across the velocity range:

$$\text{Mean fractional error}=\frac{1}{N}\sum_{V}\left(\frac{V-V'}{V}\right)\;\left(V\neq 0\right)$$ (2)

It is evident that accuracy decreases significantly as the detector centre frequency is reduced and renders the suspension increasingly homogeneous. Reducing the detector frequency further, below the lowest frequency detector used here (5 MHz), would reduce accuracy to the extent that even the detection of flow would be impossible. Note that, as discussed in section 4.1.2 below, the signal can also be bandlimited by the detector lateral field-of-view (the FWHM beamwidth in Table 1). Since the field-of-view (FOV) increases with decreasing frequency for some of the transducers in Fig. 3, this could also be responsible for the reduction in accuracy. However, this increase in FOV is modest (except for f_0_ = 5 MHz) and for some of the transducers the FOV actually reduces as the frequency decreases (e.g. f_0_ = 15 MHz and f_0_ = 25 MHz) yet the general trend of decreasing accuracy with decreasing frequency remains. This suggests that for the low concentration (5%) used in this example the FOV is not significantly bandlimiting the signal. As discussed in the following section, it is only at higher concentrations that bandlimiting due to the FOV is observed.

#### 4.1.2 Detector field-of-view (FOV)

**Fig. 4:**
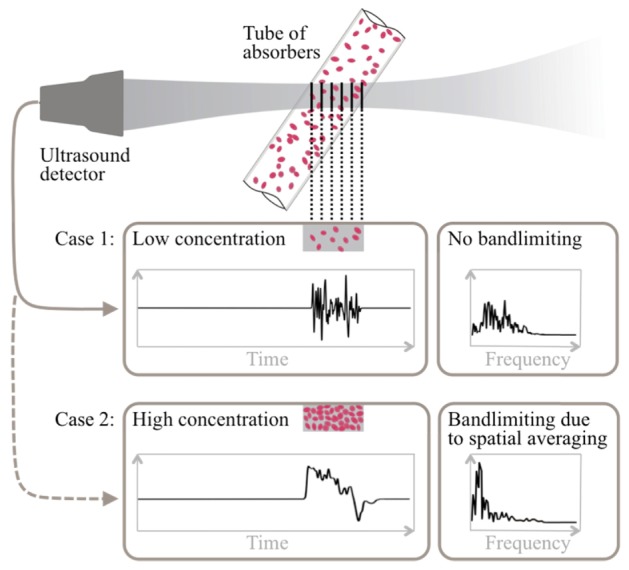
Illustration of bandlimiting due to spatial averaging over the detector field-of-view. At low concentrations, the absorbers within the tube are relatively sparsely distributed; there are rapid time-varying fluctuations in the detected photoacoustic (PA) waveform, and the acoustic frequency spectrum is broad. This permits accurate velocity measurements to be made. At high concentrations, the fluctuations in the PA waveform are smoothed out and the frequency spectrum is downshifted; the absorber distribution is perceived to be less heterogeneous and thus it is difficult to track the absorber motion and accurately measure the flow velocity.

The deleterious effect of reducing the intrinsic detector centre frequency and bandwidth on accuracy, as illustrated in Fig. 3, is perhaps unsurprising. However, as described in reference [19], there is an additional less obvious detector characteristic, namely, its lateral field-of-view (FOV), that can similarly bandlimit the signal and compromise accuracy. To illustrate this, consider the photoacoustic signal produced by a random distribution of microscopic absorbers and recorded by a focussed detector of unlimited bandwidth and a lateral FOV defined by the diameter of its focal spot as illustrated in Fig. 4

. The detected time-resolved photoacoustic signal represents a series of time-retarded projections of the initial pressure distribution p_0_ integrated over successive cross sectional surfaces at increasing depths within the focal region. At low concentrations, the absorbers are relatively sparsely distributed. The mean value of p_0_ from one cross sectional surface to the next therefore fluctuates significantly resulting in a corresponding rapid time-varying fluctuation in the detected PA waveform. This fine “structure” in the PA waveform is characteristic of a heterogeneous suspension and permits accurate velocity measurements to be made. As the concentration increases however, the fluctuations in the mean value of p_0_ gradually reduce and the structure in the PA waveform is smoothed out. The absorber distribution is then perceived as being less heterogeneous resulting in reduced measurement accuracy. Eventually, when the concentration is sufficiently high, the fine structure in the signal is lost altogether. The PA waveform is then no longer characteristic of the micron scale RBC spatial distribution but corresponds to that of a relatively large cylinder of uniform absorption. Under these conditions, the suspension is perceived as wholly homogeneous precluding even the detection of flow. A detector with a large focal spot will result in perceived homogeneity occurring at a lower concentration than a more tightly focussed detector as the former will integrate over a greater number of absorbers and thus smooth out the mean p_0_ fluctuations to a greater extent.

In the frequency domain, the above-mentioned spatial averaging effect is manifested by a downshift in the acoustic frequency spectrum: as the absorber concentration is increased, the high frequencies are suppressed whilst the low frequency content is enhanced. Note that, although the signal is bandlimited by this type of spatial averaging, it is subtly different from the bandlimiting effect imposed by the intrinsic frequency response characteristic of the detector described in section 4.1.1. In the latter case, the signal is simply low pass filtered and the energy of the signal reduced. By contrast, spatial averaging due to the detector lateral FOV produces a re-weighting or downshifting of the acoustic spectrum to lower frequencies but the total signal energy is not reduced. However, in the context of absorber heterogeneity the consequences are identical in that the higher frequencies are lost and the absorber suspension is perceived as being more homogenous thus compromising measurement accuracy.

**Fig. 5:**
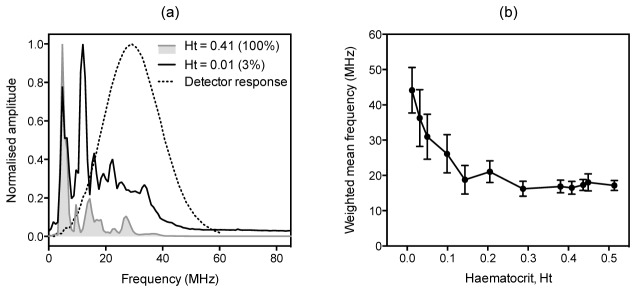
Frequency downshifting due to spatial averaging arising from the detector FOV. (a) Comparison of normalised photoacoustic signal frequency content for a high red blood cell (RBC) concentration (100% of a physiologically normal haematocrit of Ht = 0.41) and a low RBC concentration (Ht = 0.01, which corresponds to about 3% of the physiologically normal value). The normalised frequency spectra are the means of normalised fast Fourier transforms (FFTs) calculated for over 5000 PA signals, and the dotted line shows the normalised frequency response of the detector. (b) Weighted mean frequencies (WMFs) calculated from the fast Fourier transforms (FFTs) of photoacoustic (PA) signals acquired for different red blood cell (RBC) haematocrits. The WMF was calculated by summing the product of the amplitudes and the frequencies of the FFT and normalising by the sum of the amplitudes (Eq. (3)) . The data points show the mean WMF of over 1500 FFTs calculated for a set of the same number of PA signals acquired for each of the relevant RBC concentrations, and the error bars represent the standard deviation. The blood samples were taken from human volunteers, centrifuged and washed with PBS three times before diluting with PBS to give the range of haematocrits shown. In both (a) and (b) the PA signals were generated using 532 nm laser pulses and acquired using the 30 MHz focussed transducer positioned at an angle of θ = 45° relative to a 390 μm tube containing the blood suspensions.

The bandlimiting effect of spatial averaging on the acoustic frequency spectrum can be observed in Fig. 5

, which shows the frequency content of signals acquired for a high RBC concentration (100% of a physiologically normal RBC haematocrit Ht = 0.41) and a low RBC concentration (Ht = 0.01, which is approximately 3% of a physiologically realistic concentration) obtained using the 30 MHz focussed transducer (Table 1). For the 3% concentration, there is significant frequency content extending to approximately 50 MHz. For the 100% concentration, the high frequency components of the signal are significantly reduced compared to the 3% suspension with negligible frequency content beyond 30 MHz. To illustrate this downshifting behaviour more comprehensively, the weighted mean frequency (WMF) was calculated from photoacoustic spectra (comprised of *i* discrete frequency values *f_i_* each with normalised amplitude *a_i_*) obtained using samples of 12 different RBC haematocrits:

$$\text{Weighted mean frequency}=\frac{\sum_{i}a_{i}f_{i}}{\sum_{i}a_{i}}$$ (3)

These data are displayed in Fig. 5(b). It shows the mean frequency progressively decreasing as the RBC concentration increases and for haematocrits greater than about 0.2, the mean frequency remains constant at around 17 MHz. This is broadly in agreement with the results shown previously for a microsphere blood phantom [19] where the mean frequency also dropped progressively with increasing absorber concentration before reaching a plateau. The relatively low mean frequencies observed in Fig. 5(b) are perhaps surprising. Intuition based on the micron spatial scale of RBC suspensions and previous simulation studies [26,27] suggest that the frequency spectra of PA signals generated in blood should extend well beyond 100 MHz. However, these simulations either neglect the effect of the detector FOV or simulate the photoacoustic spectrum of a single RBC in which case spatial averaging is not relevant. The much lower frequencies observed in Fig. 5 are a direct consequence of the relatively large FOV (FWHM spot size ~350 µm) of the focused transducer used. This results in significant spatial averaging to the extent that it bandlimits the signal to well below the intrinsic bandwidth of both the PA source (the RBC suspension) and the detector.The results in Fig. 3 and Fig. 5 have significant implications in terms of transducer selection. As stated previously, accurate flow measurement requires that the detected signal retains sufficient high frequency content for the blood to be perceived as heterogeneous. The beneficial impact of high frequencies is evidenced by Fig. 3 which shows that significantly higher accuracy is achieved for the 30 MHz detector compared to the 5 MHz detector; in the latter case it is challenging to even detect flow since the RBC suspension is perceived as near-homogenous at this frequency. However, beyond 30 MHz, the accuracy no longer increases significantly and appears to reach a plateau. This is because the high frequency content is now limited by spatial averaging due to the lateral FOV of the detector rather than its intrinsic bandwidth so increasing the latter does not improve accuracy. The results in Fig. 3 were obtained using a low RBC concentration (Ht = 0.023, approximately 5% of a physiologically normal haematocrit). For higher concentrations, bandlimiting due to the detector FOV will be even more pronounced. As Fig. 5 shows, for a physiologically realistic concentration (Ht = 0.41), when using the 30 MHz transducer (focal spot diameter = 0.35 mm), the weighted mean frequency is less than 20 MHz and thus well below the intrinsic frequency response of the detector. Hence, for this concentration, there would be little benefit in increasing the transducer bandwidth whilst maintaining the same FOV in the expectation that more of the high frequency content conducive to accurate measurements would be retained. These results provide useful insight into the factors that govern the detected photoacoustic signal bandwidth. In particular, they explain why the frequency content in whole blood is in the tens of MHz range and thus much lower than might otherwise be expected. However, the critical question is whether the spatial heterogeneity of whole blood is on a sufficiently large scale to permit the detection of motion using acoustic detector frequencies of this order (tens of MHz). This is explored in the next section.

### 4.2 Absorber heterogeneity: measurements using whole and lysed blood

**Fig. 6:**
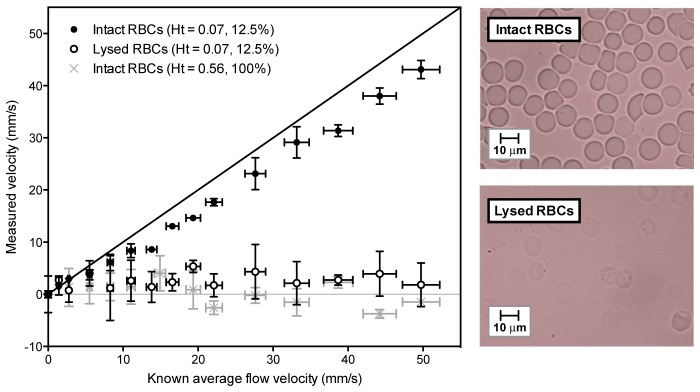
The effect of high red blood cell (RBC) concentration and RBC lysis on the accuracy of the time correlation velocity measurement. Velocity measurements were made for whole blood at a normal physiological haematocrit (grey crosses), a suspension of intact red blood cells (12.5% of a normal physiological haematocrit) in phosphate buffered saline (filled data points) and for haemolysed red blood cells (also 12.5%) in distilled water (unfilled data points). In each case, the fluid was flowing through a 400 µm tube, and the measurements were acquired using 540 nm laser pulses separated by *T* = 0.5 ms and the 30 MHz focussed transducer (θ = 45°). The length of the range gate was set such that it was greater than the tube diameter so the velocity measurement is integrated over the tube cross section. Each data point is the mean of three measurements, with a zero offset correction applied, and the vertical error bars represent the standard deviation. Microscopy images of the red blood cells are shown on the right.

The results of the previous section suggest that, for a physiologically realistic RBC concentration, the most appropriate transducer amongst those in Table 1 is the 30 MHz transducer; bandlimiting due to FOV spatial averaging means there is unlikely to be any advantage in using the 50 MHz transducer. 30 MHz is also a reasonable practical upper frequency limit in terms of achieving useful penetration depth in tissue. In order to assess whether the 30 MHz transducer is able to resolve the heterogeneity of a physiologically realistic RBC concentration it was used to detect PA signals in whole blood (RBC concentration: 5 x 10^9^ per ml; haematocrit: 0.56; haemoglobin concentration: 155 g/l) flowing through a 400 µm tube at various velocities up to 50 mm/s. In these experiments, the length of the range gate was set such that it was greater than the tube diameter so the velocity measurement is integrated over the tube cross section. The results are shown by the grey crosses in Fig. 6

and average to zero for all velocities, suggesting that RBC motion was undetectable at this physiologically realistic concentration. This may be explained by the close packing of the RBCs; perhaps after all there is insufficient absorber heterogeneity, in other words the RBC clustering at this concentration is sufficiently dense that the suspension is perceived by the detector as being homogeneous. The experiment was therefore repeated under the same conditions but using a lower concentration of RBCs, diluted with phosphate buffered saline (PBS) to give a suspension 12.5% of the original haematocrit. In this case, the concentration of the RBCs is sufficiently low that the suspension is perceived as heterogeneous and the measurements are in much closer agreement with the known flow velocity, as shown by the filled data points in Fig. 6. Finally, in order to destroy the absorber heterogeneity, the same concentration (12.5%) was tested again but in distilled water instead of PBS: the effect of the water was to haemolyse the cells which causes the haemoglobin to be released into the surrounding fluid resulting in an essentially optically homogeneous medium. As expected, the unfilled data points in Fig. 6 show that the velocity measurements average to zero across the range providing a compelling illustration of the requirement for absorber heterogeneity.

The difference in velocity measurement accuracy between the 12.5% suspension of intact RBCs compared to the lysed cell distribution shown in Fig. 6 is readily explained by the difference in absorber heterogeneity. However, the different accuracies for the two concentrations of intact RBCs (100% and 12.5%) may have an alternative explanation: it is possible that the accuracy is affected by the degree of the light penetration [19], and this is explored further in the following sub-section.

### 4.3 Light penetration

The measurements for the 12.5% suspension of intact RBCs in Fig. 6 increase linearly and show reasonable agreement with the known average flow velocity. However, it is notable that there is a consistent under-reading of this average velocity. One possible explanation is that the flow velocity profile is parabolic so the RBCs close to the tube wall are moving more slowly relative to those at the centre. Preferential absorption of light by the RBCs close to tube wall thus leads to a bias in the measurement towards the slower velocities and therefore under-reading of the average flow velocity. It is possible that under-reading also occurs for the 100% suspension for the same reason but to such an extent that the measurements appear to be consistently zero – i.e. the reduced optical penetration at this high concentration means the signal originates largely from the near static RBCs immediately adjacent to the tube wall thus biasing the measurement to zero. Of course, as noted in the previous section, the inability to detect flow could equally be a consequence of reduced heterogeneity due to the high concentration of the RBC suspension.

**Fig. 7:**
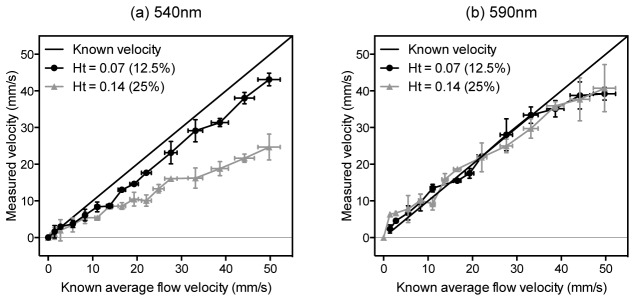
Comparison of velocity measurements made for two different red blood cell concentrations, 12.5% and 25% relative to whole blood, and for two different excitation wavelengths: 540 nm (a) and 590 nm (b). The length of the range gate was set such that it was greater than the tube diameter so the velocity measurement is integrated over the tube cross section. At 590 nm, the deeper penetration of light into the tube yields more accurate measurements. In each case, the suspensions were flowing in a 400 µm diameter tube, and the photoacoustic signals were acquired with a 30 MHz focussed transducer (θ = 45°). The measurements were acquired using laser pulses separated by *T* = 0.5 ms. Each data point is the mean of three measurements, with a zero offset correction applied, and the vertical error bars represent the standard deviation.

In order to distinguish between the effect of light attenuation from that of absorber heterogeneity, the wavelength of the excitation light was varied. By exploiting the spectral dependence of haemoglobin absorption, this enables the optical penetration within the tube to be varied whilst maintaining constant heterogeneity. Velocity measurements were made with blood diluted with PBS to give concentrations of 12.5% and 25%, first using a wavelength of 540 nm and second using 590 nm where the absorption coefficient of blood is nearly four times smaller (see Appendix, Fig. 11). Figure 7(a)

shows the velocity measurements at 540 nm: both concentrations demonstrate under-reading of the average flow velocity, but the under-reading is greater for the 25% concentration, which could be explained either by the reduced heterogeneity or by the increased light attenuation or a combination of both. As shown in Fig. 7(b), at a wavelength of 590 nm, the accuracy of the measurements made with both concentrations is excellent with very close agreement to the known velocity. This suggests that the greater penetration of light at the longer wavelength yields measurements that are less biased towards the slower velocities at the edge of the tube and therefore more accurately represent the average flow velocity across the tube. It also suggests that for a 25% RBC concentration, there is clearly sufficient absorber heterogeneity and the intrinsic detector frequency response characteristics and FOV are not limiting factors in this respect.

**Fig. 8:**
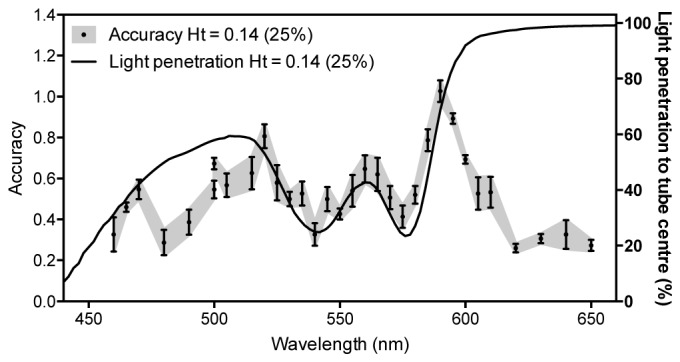
Variation of measurement accuracy with the wavelength of the excitation lasers. The length of the range gate was set such that it was greater than the tube diameter so the velocity measurement is integrated over the tube cross section. The solid line represents the percentage of light (right axis) that penetrates into the centre of the tube (diameter: 390 µm), calculated from the absorption coefficient spectrum of oxyhaemoglobin (see Appendix, Fig. 11). The data points correspond to the accuracy (left axis) of velocity measurements made for a blood suspension (25% relative to whole blood) flowing at 15 mm/s. For each wavelength, the accuracy (1-fractional error) was calculated for the mean of five velocity measurements, and the vertical error bars correspond to the standard error.

**Fig. 9:**
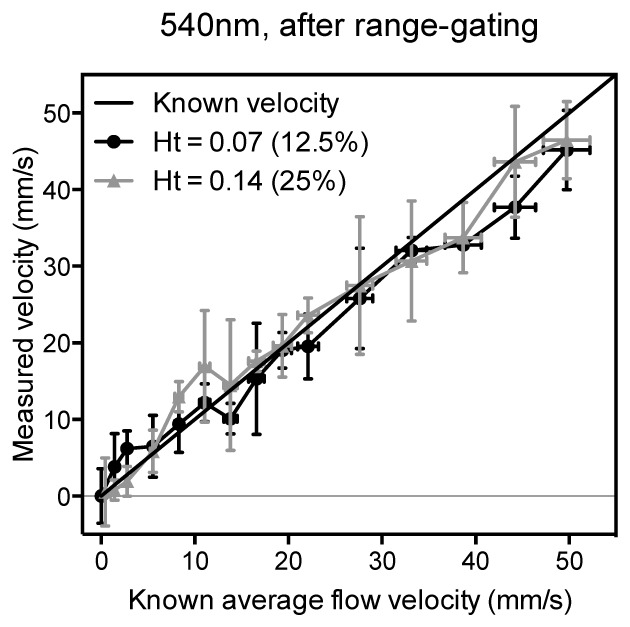
Comparison of the accuracy of velocity measurements made for two different red blood cell concentrations, 12.5% and 25% relative to whole blood, and a wavelength of 540 nm. The data are those shown in Fig. 7(a) but after time-windowing to remove the bias towards low velocities due to the greater absorption at the edge of the tube.

The improvement in accuracy using 590 nm compared to 540 nm is more significant for the 25% concentration and so this concentration was used to investigate the effect of different wavelengths further. Five velocity measurements were made for the suspension flowing at 15 mm/s, and at various wavelengths ranging from 460 nm to 650 nm. The results are shown in Fig. 8

and compared with the proportion of light penetrating to the centre of the tube, which varies with wavelength according to the absorption spectrum (see Fig. 11). For wavelengths between 520 nm and 595 nm there is a clear relationship between the measurement accuracy and the light penetration, and it is notable that at most wavelengths there is significant under-reading of the average flow velocity (accuracy is a fractional error less than 1.0). The best accuracies are at the longer wavelengths, particularly at 590 nm and 595 nm, where the light penetration is greater than at shorter wavelengths, and therefore the measured velocities more closely represent the average flow velocity. For wavelengths beyond 595 nm the measurement accuracy deteriorated due to the low SNR, exacerbated not only by the low absorption coefficient but also by the reduced laser output at these wavelengths.The results in Fig. 7 and Fig. 8 were obtained by setting the length of the range gate such that it was greater than the tube diameter so the velocity measurement is integrated over the tube cross section. This inevitably gives a weighting to the larger amplitude part of the signal arising from the slower moving absorbers near the edge of the tube where most of the light is absorbed. This can result in under-reading of the average flow velocity, particularly for the more strongly absorbed shorter wavelengths. It has been shown in previous work [19] that selection of a range gate corresponding to a deeper region within the tube can remove the bias towards lower velocities due to this effect, and thereby improve the measurement accuracy. In order to determine the efficacy of this approach for shorter wavelengths where the light penetration is limited, it was applied to the photoacoustic signals acquired for the two concentrations at 540 nm, and the results are shown in Fig. 9

. Comparison with Fig. 7(a) shows that this removes the bias towards the slower velocities and thus the measurements more accurately correlate with the known average flow velocities. This improvement is particularly notable for the 25% concentration where the mean fractional error is initially about 37% but is reduced to about 2% after range-gating is applied.The results in Fig. 7, Fig. 8 and Fig. 9 highlight two important findings. First, it is clear from Fig. 8 that, due to the spectral dependence of the blood absorption coefficient, longer wavelengths allow deeper light penetration and therefore the measured velocities more accurately match the known average flow velocity; this is also supported by the data in Fig. 7. However, Fig. 9 shows that even at a shorter, highly attenuated wavelength of light (540 nm) it is still possible to compensate for the bias towards the slower velocities at the edge of the tube by analysing time windows of the photoacoustic signal corresponding to a deeper region. Second, the ability to achieve accurate measurements using the 12.5% and 25% concentrations which are in reasonable proximity to physiologically realistic haematocrits suggests that the heterogeneity of whole blood may prove not to be a wholly confounding factor. Following these findings, it is therefore now pertinent to consider whether range-gating in this way could be employed to improve on the results obtained in Fig. 6 which showed that at a physiologically realistic concentration it was not possible to detect flow

**Fig. 10:**
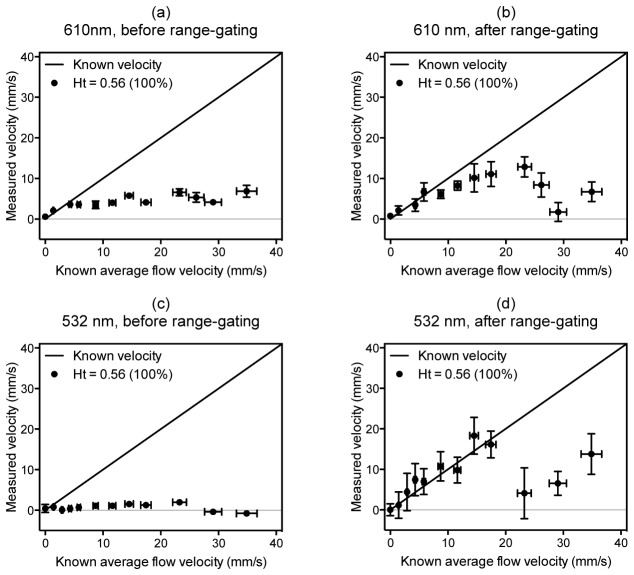
Velocities measured for fresh, whole blood at a physiologically realistic concentration flowing in a tube of diameter 390 μm, and illuminated first with 610 nm light (a,b) and second with 532 nm light (c,d). The data were acquired with a laser pulse separation of *T* = 0.5 ms and a 30 MHz focused transducer (θ = 45°). In (a) and (c) the velocities were calculated by cross-correlating entire photoacoustic waveforms, whereas the velocities in (b) and (d) correspond to a single waveform segment selected by time-windowing. In (a) and (b) the data points are the mean of 5 velocity measurements and the vertical error bars represent the standard error; in (c) and (d) the data points are the mean of 20 velocity measurements and the vertical error bars represent the standard error.

**Fig. 11:**
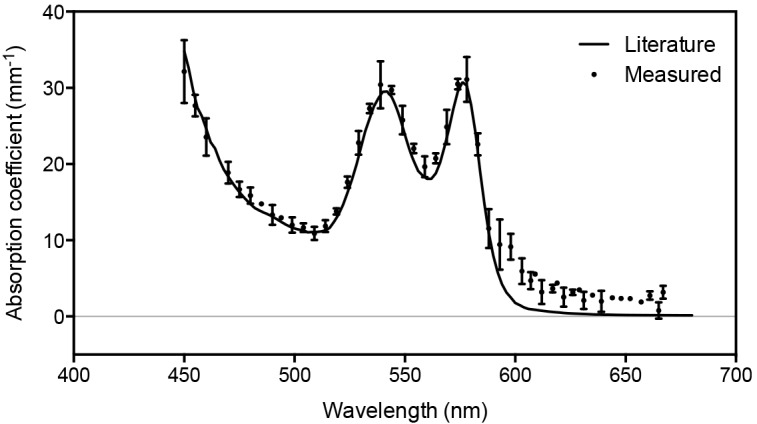
Absorption coefficients for oxyhaemoglobin (solid line) plotted over a wavelength range of 450 nm to 680 nm [28]. The data points were obtained by fitting an exponential function [29] to photoacoustic waveforms detected in whole blood. There is excellent agreement between the measured and literature values for wavelengths below about 590 nm; for longer wavelengths the exponential fit becomes less reliable due to the low amplitude photoacoustic signal.

Measurements were therefore made using the “100%” RBC concentration (whole blood at a physiologically realistic concentration of 5 x 10^9^ per ml, Ht = 0.56) at two different wavelengths, 610 nm and 532 nm, with and without range-gating. The results are plotted in Fig. 10

. Figure 10(a) shows that without range-gating, even at a long wavelength (610 nm), where about 85% of the light penetrates to the centre of the tube, measurement accuracy is poor. Despite this, the measurements are not uniformly zero suggesting that it is possible to at least detect the motion of the RBC suspension. This implies that the suspension is not being perceived by the detector as wholly homogenous and that the light penetration may still be a contributing factor to the poor accuracy. To test this hypothesis, the signals were range-gated to select time windows corresponding to deeper regions in the tube and the velocities re-estimated. These results are displayed in Fig. 10(b) and it can be seen that the accuracy is significantly improved particularly below 20 mm/s.

The measurements at 532 nm further support this interpretation. At this wavelength, where only 1% of the light penetrates to the centre of the tube, detection of flow appears impossible (Fig. 10(c)) without range-gating. However, when range-gating is applied, the average flow velocity can be recovered with reasonable accuracy for speeds less than 20 mm/s, as shown in Fig. 10(d). This was achieved by taking many repeated measurements (n = 20), hence the larger error bars, whereas for 610 nm fewer measurements were adequate (n = 5). The difficulty in making accurate measurements at velocities greater than 20 mms/ is likely to be due to de-correlation as the RBCs change their relative positions and move out of the transducer focal region, but this could potentially be reduced by using a laser pulse separation shorter than the current value of *T* = 0.5 ms.

These results illustrate that although limited light penetration compromises measurement accuracy, it can be mitigated by appropriate selection of the range gate. Perhaps most notably, the results show that measurement can be made in whole blood of a physiologically realistic concentration. This suggests that blood is sufficiently heterogeneous to be resolved by detectors with a bandwidth of several tens of MHz.

## 5. Discussion

This study has demonstrated that flow measurements can be made using time-correlation AR-PAF in whole blood at physiologically realistic concentrations. In arriving at this result, new insight into the conditions required for accurate measurements has been acquired. This has revealed two critical factors that require consideration for the measurement of flow, namely, absorber heterogeneity and light penetration.

As discussed in sections 4.1 and 4.2, the detection and measurement of flow requires the RBC suspension to be optically heterogeneous. Figure 6 provides a compelling illustration of this requirement, since no motion could be detected after rupturing of the RBCs released haemoglobin into the plasma resulting in an optically homogeneous medium. Heterogeneity in this context however can only be described relative to the minimum detectable wavelength which depends on the detector characteristics. The most obvious consideration is the intrinsic transducer frequency response, with lower frequency transducers perceiving reduced heterogeneity resulting in reduced velocity measurement accuracy. Less obviously, the signal can also be bandlimited and accuracy similarly compromised as a result of the spatial averaging effect imposed by the detector FOV. The influence of both characteristics are illustrated by the results in Fig. 3 and Fig. 5 respectively, and both confirm previous work using absorbing microsphere based flow phantoms [19]. The key implication is that heterogeneity cannot be considered in terms of the intrinsic detector centre frequency and bandwidth alone; in addition, spatial averaging due to the detector FOV must be considered as it can dramatically bandlimit the signal thus reducing the perceived heterogeneity and compromising accuracy.

With the insight provided by the results in Fig. 3 and Fig. 5 we can now begin to consider the transducer requirements for successful flow measurements in terms of intrinsic frequency response and FOV. Both are inextricably linked. Using a very wide intrinsic detector bandwidth extending to several hundred MHz with the intention of resolving the fine granularity exhibited by blood would serve little purpose if the signal is bandlimited to a few tens of MHz by spatial averaging due to a large detector FOV. This point is illustrated by Fig. 5(b), which shows that the weighted mean frequency decreases to a plateau around 20 MHz as the haemotocrit approaches physiologically realistic levels. In this case, there would be little advantage to increasing the transducer frequency (30 MHz) for the same FOV. The question then is whether the heterogeneity of blood is on a spatial scale that corresponds to this frequency. If it is, then flow measurement should be possible. At first sight, this might seem unlikely. If we regard whole blood as a random non-aggregated distribution of individual red blood cells at a physiologically realistic concentration of 4 x 10^9^ cells/ml, this would imply a mean RBC separation of 2.5 µm which corresponds to a frequency of approximately 600 MHz. A transducer with a bandwidth of a few tens of MHz as used in this study would perceive such a suspension as homogeneous. Indeed, such a transducer would perceive even a 25% concentration suspension as near homogeneous. However the results in Fig. 7 and Fig. 9 show that excellent accuracy is possible for this concentration and Fig. 10 shows that flow detection is possible even using a 100% RBC suspension. This suggests that blood is significantly more heterogeneous than the above model of a random non-aggregated distribution of RBC implies. This is perhaps not surprising. It is well known that clustering, aggregation and the formation of RBC rouleaux can occur in whole blood. This could result in a coarser absorber distribution than might otherwise be expected and one that is sufficiently heterogeneous to be resolved by transducers with frequencies in the tens of MHz range as used in this study.

What then are the optimum transducer characteristics? As noted above, flow measurements in whole blood at a physiologically realistic concentration were achieved using the 30 MHz transducer with 0.35 mm spot size. However, accuracy, resolution and repeatability were lower than achieved with the lower concentrations (5%, 12.5% and 25%) suggesting insufficient perceived heterogeneity may still be a negative influence. It may be possible to address this by reducing the detector spot size in order to reduce the bandlimiting due to spatial averaging. Reducing the spot size however requires reducing *T* which decreases accuracy, resolution and the maximum detectable velocity. A practical lower limit of around 50 µm is estimated before the technique would be excessively compromised in this respect. There may also be benefit in increasing the transducer centre frequency and bandwidth although this would incur a cost in tissue penetration depth – for example, acoustic attenuation in tissue at 30 MHz for a 5 mm pathlength is approximately 15 dB so increasing the detector frequency significantly will reduce penetration depth further, potentially to depths only marginally greater than achievable using the optical resolution mode (less than about 1 mm). Whilst deeper penetration would be desirable, the results in Fig. 3 suggest that accurate AR-PAF measurements using detectors sensitive only to a few MHz as required for centimetre scale penetration depths may be challenging since blood may be perceived as near homogeneous at these frequencies. This does raise the question as to why pulsed Doppler ultrasound flowmetry works *in vivo* using detectors in the low MHz frequency range, even though it would seem to have a heterogeneity requirement similar to that of AR-PAF. One explanation may be related to the role of speckle. This arises from the presence of sub-wavelength spatial heterogeneities and is dominant in ultrasound techniques but appears to be less so in photoacoustic methods, although the reason for the latter remains incompletely understood.

Whilst limited heterogeneity can compromise measurement accuracy in whole blood, SNR may also be a factor. Fundamentally, there is a trade-off between light penetration and SNR. Figure 7, Fig. 8 and Fig. 9 show that limited light penetration biases the velocity measurements towards under-reading. Measurement accuracy is improved by increasing the penetration depth which can be achieved either by employing less attenuated wavelengths of light, or by using range-gating to select deeper signal segments. However, by the same token, both strategies reduce the SNR. In this study, accurate measurements were possible using wavelengths less than 610 nm to irradiate a single blood filled tube immersed in water and excited by laser pulses close to the MPE. An *in vivo* measurement of even a superficial blood vessel is likely to result in lower SNR due to higher optical and acoustic attenuation. Although such an SNR may still be more than adequate to detect the vessel, it needs to be sufficient to resolve the very small fluctuations in the signal on to which the velocity information is encoded. In this sense AR-PAF is particularly sensitive to SNR. It remains to be determined whether the limiting factor for making accurate, high resolution blood velocity measurements is related to heterogeneity or to this critical balance between light penetration and SNR.

## 6. Conclusion

This work has demonstrated for the first time that the velocity of whole blood (100% of a physiologically realistic haematocrit) can be estimated using AR-PAF. Moreover it has been shown that the spatial heterogeneity of whole blood is sufficient to permit flow detection using an ultrasound detector with a centre frequency and bandwidth on a scale of tens of MHz and a field-of-view (FOV) of hundreds of micrometres. This somewhat surprising result is likely to be due to the aggregated nature of whole blood which results in significantly greater spatial heterogeneity than from a non-aggregated random distribution of RBCs thereby permitting flow measurement. Measurement accuracy depends on the ability of the transducer to resolve the RBC distribution and this depends critically on both the detector frequency and the FOV, which in this case were 30 MHz and 350 µm respectively. It is likely that there is some further scope to improve accuracy over the results reported in this study via a modest increase in transducer frequency and a commensurate reduction in FOV. However a significant increase in the transducer frequency would risk excessively reducing penetration depth in tissue. Accuracy is also influenced by limited light penetration. Choosing the wavelength or applying range-gating in order to increase the depth of the measurement improves accuracy but this inevitably compromises SNR, which in turn can have detrimental effects on accuracy and resolution.

The results obtained in this study are encouraging in that, whilst they confirm that the requirement for optical absorption heterogeneity poses a fundamental limit, they also suggest that there is sufficient heterogeneity in whole blood to enable *in vivo* measurements providing the transducer characteristics are carefully chosen. Detectors with bandwidths in the tens of MHz frequency range can perceive adequate heterogeneity but would probably limit velocity measurements to depths of less than 5 mm in tissue. A further limit to the penetration depth is imposed by the SNR, which will be reduced when making measurements *in vivo* and at the longer wavelengths required for increased penetration depth. These factors suggest that scaling AR-PAF to the upper range of penetration depths (several centimetres) accessible by photoacoustic imaging techniques may be challenging and could require additional insight for success. Nevertheless, the ability to make blood velocity measurements in the microvasculature to even mm scale depths as suggest by the current study would provide a valuable complement to existing flowmetry techniques for studying microcirculatory abnormalities associated with cancer, diabetes and cardiovascular disease.

## References

[1] Evans D. H. McDicken W. N., *Doppler Ultrasound: Physics, Instrumentation, and Signal Processing*, 2nd ed. (Wiley, 2000).

[2] Golan L. Yeheskely-Hayon D. Minai L. Dann E. J. Yelin D., “Noninvasive imaging of flowing blood cells using label-free spectrally encoded flow cytometry,” Biomed. Opt. Express 3(6), 1455–1464 (2012). 10.1364/BOE.3.001455 22741090 PMC3370984

[3] Cinotti E. Gergelé L. Perrot J. L. Dominé A. Labeille B. Borelli P. Cambazard F., “Quantification of capillary blood cell flow using reflectance confocal microscopy,” Skin Res. Technol. 20(3), 373–378 (2014). 10.1111/srt.12128 24506277

[4] de Carlo T. E. Romano A. Waheed N. K. Duker J. S., “A review of optical coherence tomography angiography (OCTA),” Int. J. Retin. Vitr. 1(1), 5 (2015). 10.1186/s40942-015-0005-8 PMC5066513 27847598

[5] Liu G. Lin A. J. Tromberg B. J. Chen Z., “A comparison of Doppler optical coherence tomography methods,” Biomed. Opt. Express 3(10), 2669–2680 (2012). 10.1364/BOE.3.002669 23082305 PMC3469988

[6] Vennemann P. Lindken R. Westerweel J., “In vivo whole-field blood velocity measurement techniques,” Exp. Fluids 42(4), 495–511 (2007). 10.1007/s00348-007-0276-4

[7] Brunker J. Beard P., “Pulsed photoacoustic Doppler flowmetry using time-domain cross-correlation: accuracy, resolution and scalability,” J. Acoust. Soc. Am. 132(3), 1780–1791 (2012). 10.1121/1.4739458 22978905

[8] Chen S. L. Ling T. Huang S. W. Won Baac H. Guo L. J., “Photoacoustic correlation spectroscopy and its application to low-speed flow measurement,” Opt. Lett. 35(8), 1200–1202 (2010). 10.1364/OL.35.001200 20410966 PMC2859458

[9] Sheinfeld A. Gilead S. Eyal A., “Photoacoustic Doppler measurement of flow using tone burst excitation,” Opt. Express 18(5), 4212–4221 (2010). 10.1364/OE.18.004212 20389434

[10] Sheinfeld A. Gilead S. Eyal A., “Simultaneous spatial and spectral mapping of flow using photoacoustic Doppler measurement,” J. Biomed. Opt. 15(6), 066010 (2010). 10.1117/1.3509113 21198184

[11] van den Berg P. J. Daoudi K. Steenbergen W., “Review of photoacoustic flow imaging: its current state and its promises,” Photoacoustics 3(3), 89–99 (2015). 10.1016/j.pacs.2015.08.001 26640771 PMC4595496

[12] Yao J. Wang L. V., “Transverse flow imaging based on photoacoustic Doppler bandwidth broadening,” J. Biomed. Opt. 15(2), 021304 (2010). 10.1117/1.3339953 20459226 PMC2857455

[13] Chen S.-L. Xie Z. Carson P. L. Wang X. Guo L. J., “In vivo flow speed measurement of capillaries by photoacoustic correlation spectroscopy,” Opt. Lett. 36(20), 4017–4019 (2011). 10.1364/OL.36.004017 22002371 PMC3319062

[14] Liang J. Zhou Y. Winkler A. W. Wang L. Maslov K. I. Li C. Wang L. V., “Random-access optical-resolution photoacoustic microscopy using a digital micromirror device,” Opt. Lett. 38(15), 2683–2686 (2013). 10.1364/OL.38.002683 23903111 PMC3784350

[15] Wang L. Maslov K. Wang L. V., “Single-cell label-free photoacoustic flowoxigraphy in vivo,” Proc. Natl. Acad. Sci. U.S.A. 110(15), 5759–5764 (2013). 10.1073/pnas.1215578110 23536296 PMC3625281

[16] Yao J. Gilson R. C. Maslov K. I. Wang L. Wang L. V., “Calibration-free structured-illumination photoacoustic flowgraphy of transverse flow in scattering media,” J. Biomed. Opt. 19(4), 046007 (2014). 10.1117/1.JBO.19.4.046007 24718385 PMC3980702

[17] Zhang R. Wang L. Yao J. Yeh C.-H. Wang L. V., “In vivo optically encoded photoacoustic flowgraphy,” Opt. Lett. 39(13), 3814–3817 (2014). 10.1364/OL.39.003814 24978744 PMC4165860

[18] Zhou Y. Liang J. Maslov K. I. Wang L. V. L., “Calibration-free in vivo transverse blood flowmetry based on cross correlation of slow time profiles from photoacoustic microscopy,” Opt. Lett. 38(19), 3882–3885 (2013). 10.1364/OL.38.003882 24081077 PMC3831365

[19] Brunker J. Beard P., “Acoustic resolution photoacoustic Doppler velocimetry in blood-mimicking fluids,” Sci. Rep. 6, 20902 (2016). 10.1038/srep20902 26892989 PMC4759580

[20] van den Berg P. J. Daoudi K. Steenbergen W., “Pulsed photoacoustic flow imaging with a handheld system,” J. Biomed. Opt. 21(2), 026004 (2016). 10.1117/1.JBO.21.2.026004 26857470

[21] O’Rourke M. F. Nichols W. W. Vlachopoulos C., *Mcdonald’s Blood Flow in Arteries: Theoretical, Experimental and Clinical Principles*, 6th ed. (Taylor & Francis Ltd, 2011).

[22] Sagar S. M. Klassen G. A. Barclay K. D. Aldrich J. E., “Tumour blood flow: measurement and manipulation for therapeutic gain,” Cancer Treat. Rev. 19(4), 299–349 (1993). 10.1016/0305-7372(93)90009-G 7693345

[23] P. C. Beard, “Flow velocity measurements,” in *UK Patent Application* (WO 03/039364, 2001).

[24] Zhang E. Laufer J. Beard P., “Backward-mode multiwavelength photoacoustic scanner using a planar Fabry-Perot polymer film ultrasound sensor for high-resolution three-dimensional imaging of biological tissues,” Appl. Opt. 47(4), 561–577 (2008). 10.1364/AO.47.000561 18239717

[25] Olympus, “Ultrasonic Transducers Technical Notes,” http://www.olympus-ims.com/en/resources/white-papers/ultrasonic-transducer-technical-notes/.

[26] Strohm E. M. Berndl E. S. L. Kolios M. C., “Probing red blood cell morphology using high-frequency photoacoustics,” Biophys. J. 105(1), 59–67 (2013). 10.1016/j.bpj.2013.05.037 23823224 PMC3699781

[27] Hysi E. Saha R. K. Kolios M. C., “Photoacoustic ultrasound spectroscopy for assessing red blood cell aggregation and oxygenation,” J. Biomed. Opt. 17(12), 125006 (2012). 10.1117/1.JBO.17.12.125006 23235833

[28] Prahl S., “Optical absorption of haemoglobin,” http://omlc.org/spectra/hemoglobin/.

[29] Laufer J. Zhang E. Beard P., “Evaluation of Absorbing Chromophores Used in Tissue Phantoms for Quantitative Photoacoustic Spectroscopy and Imaging,” IEEE J. Sel. Top. Quantum Electron. 16(3), 600–607 (2010). 10.1109/JSTQE.2009.2032513

